# Immersive Virtual Reality Use in Medical Intensive Care: Mixed Methods Feasibility Study

**DOI:** 10.2196/62842

**Published:** 2024-08-09

**Authors:** Brian W Locke, Te-yi Tsai, C Mahony Reategui-Rivera, Aileen S Gabriel, Aref Smiley, Joseph Finkelstein

**Affiliations:** 1 Division of Respiratory, Critical Care, and Occupational Pulmonary Medicine Department of Internal Medicine University of Utah Salt Lake City, UT United States; 2 Department of Pulmonary and Critical Care Intermountain Medical Center Murray, UT United States; 3 Department of Biomedical Informatics University of Utah Salt Lake City, UT United States

**Keywords:** immersive virtual reality, intensive care unit, distraction therapy, virtual reality, mixed methods, feasibility study, semistructured interview, therapy, therapist, critical illness, critically ill, adult, patient acceptance, user experience, games for health, serious games, gamification

## Abstract

**Background:**

Immersive virtual reality (VR) is a promising therapy to improve the experience of patients with critical illness and may help avoid postdischarge functional impairments. However, the determinants of interest and usability may vary locally and reports of uptake in the literature are variable.

**Objective:**

The aim of this mixed methods feasibility study was to assess the acceptability and potential utility of immersive VR in critically ill patients at a single institution.

**Methods:**

Adults without delirium who were admitted to 1 of 2 intensive care units were offered the opportunity to participate in 5-15 minutes of immersive VR delivered by a VR headset. Patient vital signs, heart rate variability, mood, and pain were assessed before and after the VR experience. Pre-post comparisons were performed using paired 2-sided *t* tests. A semistructured interview was administered after the VR experience. Patient descriptions of the experience, issues, and potential uses were summarized with thematic analysis.

**Results:**

Of the 35 patients offered the chance to participate, 20 (57%) agreed to partake in the immersive VR experience, with no difference in participation rate by age. Improvements were observed in overall mood (mean difference 1.8 points, 95% CI 0.6-3.0; *P*=.002), anxiety (difference of 1.7 points, 95% CI 0.8-2.7; *P*=.001), and pain (difference of 1.3 points, 95% CI 0.5-2.1; *P*=.003) assessed on 1-10 scales. The heart rate changed by a mean of –1.1 (95% CI –0.3 to –1.9; *P*=.008) beats per minute (bpm) from a baseline of 86.1 (SD 11.8) bpm and heart rate variability, assessed by the stress index (SI), changed by a mean of –5.0 (95% CI –1.5 to –8.5; *P*=.004) seconds^–2^ from a baseline SI of 40.0 (SD 23) seconds^–2^. Patients commented on the potential for the therapy to address pain, lessen anxiety, and facilitate calmness. Technical challenges were minimal and there were no adverse effects observed.

**Conclusions:**

Patient acceptance of immersive VR was high in a mostly medical intensive care population with little prior VR experience. Patients commented on the potential of immersive VR to ameliorate cognitive and emotional symptoms. Investigators can consider integrating minimally modified commercial VR headsets into the existing intensive care unit workflow to further assess VR’s efficacy for a variety of endpoints.

## Introduction

Patients with critical illness experience many noxious sensations, stress, and restricted mobility while being treated in intensive care units (ICUs). There is a burgeoning evidence base demonstrating a loss in mental, emotional, and physical functioning after critical illness. Approaches to improve the experience of critical illness and functional outcomes after hospitalization are needed.

Immersive virtual reality (VR) has been proposed as a promising tool to address these issues [1,2]. Immersive VR often involves the use of a headset to project the viewer into an interactive artificial environment that elicits the feeling of embodiment in the artificial environment [3]. Preliminary work assessing the efficacy of immersive VR for physical and cognitive mobilization [4-8], sleep [9], distraction from pain [10,11], and mood [12,13] has been performed, with many further trials ongoing.

A major challenge to applying the results from prior studies of VR is that there are numerous permutations of how, when, and for whom immersive VR might be used. For example, the equipment [14], particular VR experience [14,15], and clinical purpose [3] may all vary. Potential barriers that might influence whether VR is accepted could be specific to the setting, providers, and patients for which the VR is being used [16]. Accordingly, widely variable uptake has been reported in prior studies [6,17,18]. Thus, it is difficult to infer, from the current literature, how acceptable VR might be in a particular situation.

In this study, we evaluated the feasibility of an immersive VR experience for patients admitted to one of 2 ICUs at a single institution. We hypothesized that patients with critical illness in the ICU would be interested in experiencing immersive VR; patients and staff would encounter minimal barriers to its use; and that VR usage would be associated with improvements in qualitative and quantitative accounts of mood, anxiety, and well-being.

## Methods

### Study Design and Setting

We performed a prospective, mixed methods, nonrandomized feasibility study of patients with critical illness using immersive VR headsets. The study was conducted in 2 ICUs (a 25-bed medical ICU and a 16-bed cancer-specialty ICU that cares for both medical and surgical critically ill patients with cancer) at a single institution in Salt Lake City, Utah, United States. Reporting follows the STROBE (Strengthening the Reporting of Observational Studies in Epidemiology) guidance for observational research (Multimedia Appendix 1)[19].

### Ethical Considerations

The study was approved by the University of Utah Institutional Review Board (00170975). Patients were individually consented with a waiver of the requirement for written documentation of consent. Patients were not offered compensation for participation. Deidentified data were recorded in case report forms that were later digitized for analysis.

### Recruitment

Patients who were potentially eligible for study inclusion were identified by attending physicians after daily rounds on days when study staff were available for enrollment. To be included, patients needed to be 18 years or older, admitted to the ICU, free from delirium [20] (as assessed by their providers, nurses, and able to pass an additional attention screen), and able to consent on their own behalf. Exclusion criteria included severe visual or auditory impairments (eg, legal blindness or deafness), isolation precautions for infection, recent condition that could be potentially exacerbated by VR (eg, seizure, uncontrolled nausea, traumatic brain injury, history of psychosis, or admission for a mental health crisis), or other craniofacial injury prohibiting headset use. Additionally, patients under current use of an orofacial mask to deliver positive airway pressure ventilation were excluded as the mask precluded headset use; however, intubated patients, patients receiving high-flow nasal cannula, and patients receiving oxygen via a regular face mask were eligible to participate.

After patients were identified as potential candidates, nursing staff were approached to identify any conflicting patient-care tasks (eg, physical therapy or travel for diagnostic testing or procedures). Patients were then approached about whether they were interested in trying an experimental immersive VR therapy. Patient demographics, reasons for admission, and comorbidities were assessed by chart review of clinical notes. All patients who were identified as potential research participants by their attending physicians were screened for inclusion and approached if eligible.

### Experimental Protocol

**Figure 1:**
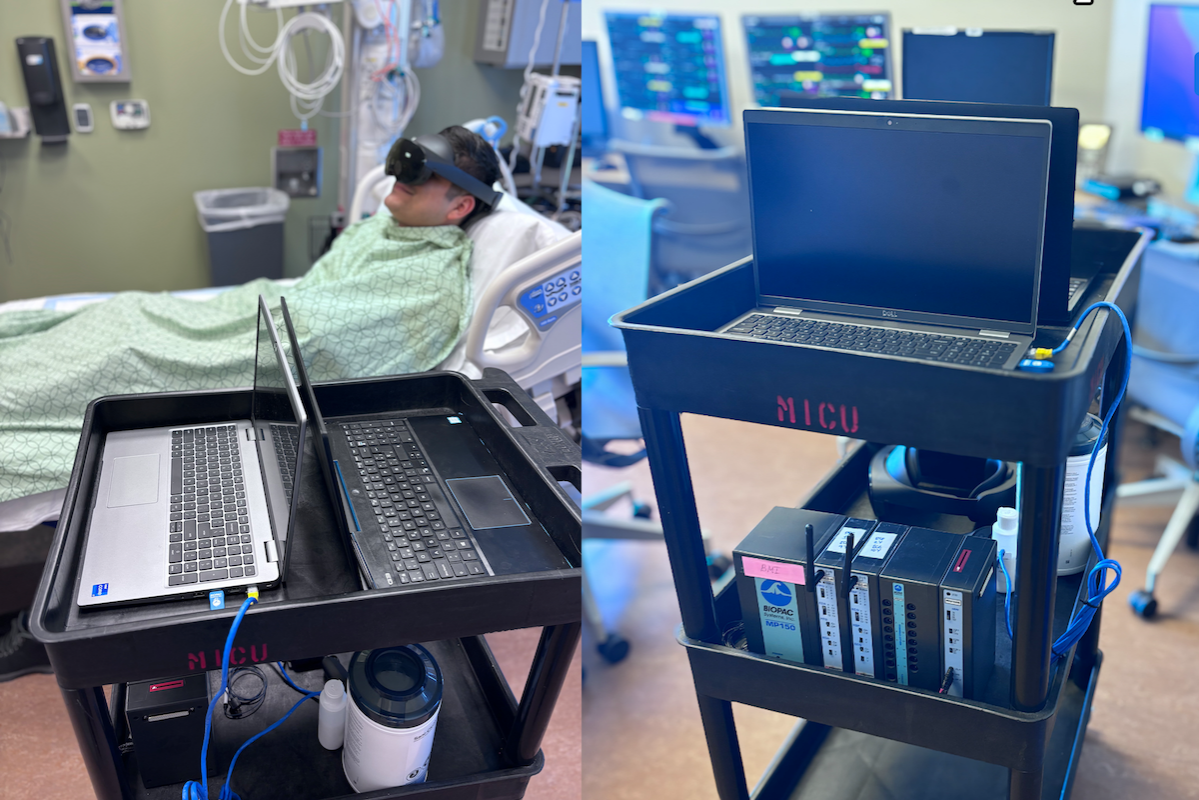
Experimental setup, as demonstrated by study personnel. Two laptops were used to ensure the adequacy of monitoring, project the real-time virtual reality experience of the patient to troubleshoot any technical issues or adverse effects, and to transcribe interview responses after the experience. The Meta Quest headset was chosen out of several possibilities owing to the lack of fabric components to facilitate cleaning after use.

Participating patients were presented with 3 visual analog scales (1-10) asking them to rate their current overall well-being, level of anxiety, and level of pain. Preintervention heart rate signals were recorded using a BIOPAC MP160 system for 5 minutes prior to VR initiation (Figure 1).

**Figure 2:**
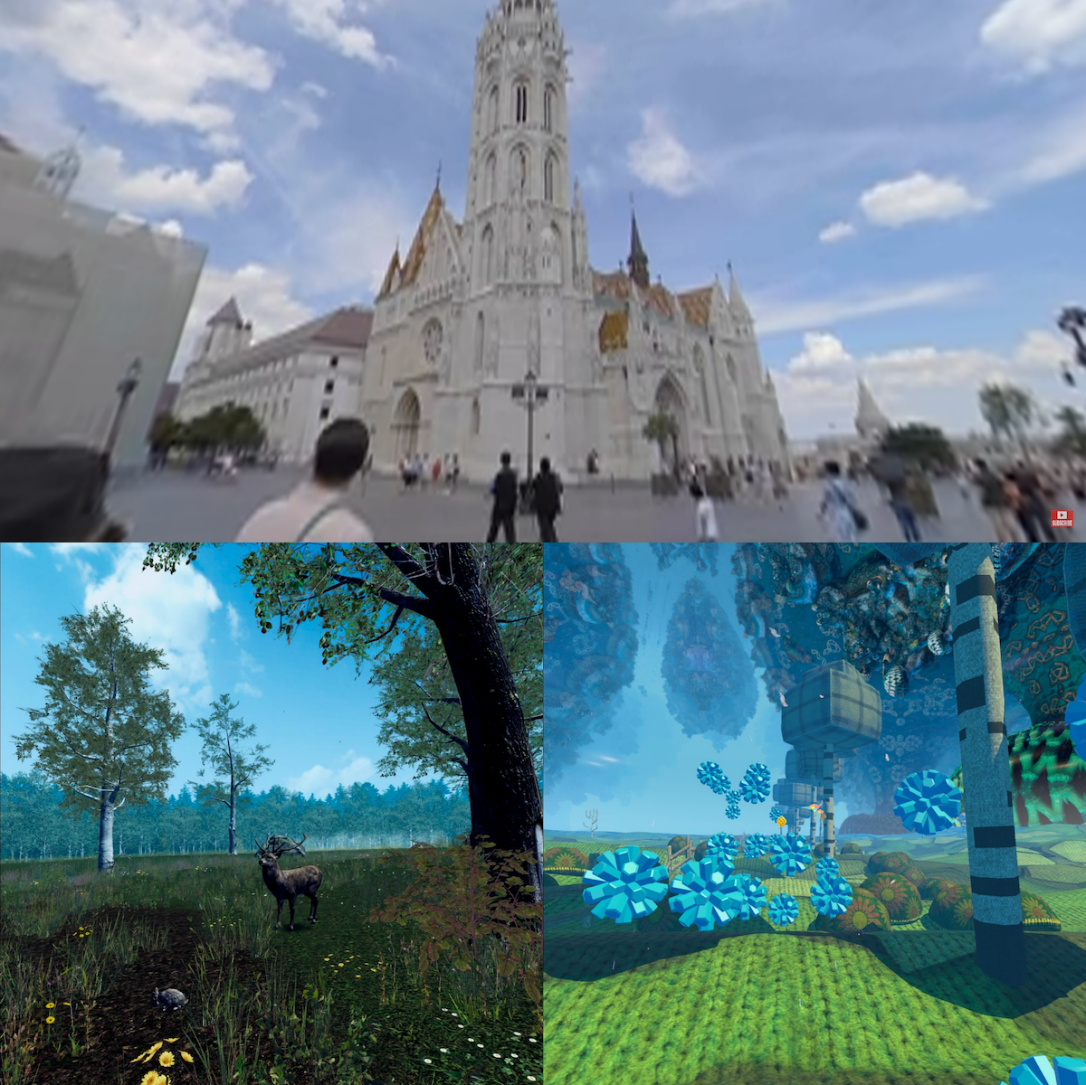
Screenshots from the virtual reality experiences. Patients chose between one of the following experiences: YouTube VR (Google LLC; top panel), Nature Treks VR (GreenerGames; bottom left panel), or TRIPP (TRIPP Inc; bottom right panel).

Patients were offered 1 of 3 commercially available VR experiences delivered by a Meta Quest Pro headset (Meta): an urban travel experience (YouTube VR; Google LLC), a nature experience (Nature Treks VR; GreenerGames), or a synthetic landscape experience (TRIPP). In all 3 scenarios, the experiences involved passive exploration of the environment and did not involve use of the hand controllers (Figure 2). Patients planned to use the VR headset for at least 5 minutes, with an option to continue for up to 15 minutes if desired. Physiologic recording was continued for 5 minutes after VR use was complete.

After completing the VR experience, 5 minutes of postintervention vital signs recording and visual analog scale assessments of well-being, anxiety, and pain were administered. These scales were modeled on the Visual Analog Mood Scale [21], Numeric Visual Analog Anxiety Scale [22], and Verbal Numerical Rating Scale for pain [23], respectively (Multimedia Appendix 2).

Lastly, a semistructured qualitative interview was conducted to elicit feedback. A moderator guide (Multimedia Appendix 3) consisting of open-ended questions aimed at understanding the patient’s overall experience using the VR headset was administered by a trained researcher. The responses for each participant were transcribed concurrently by 2 researchers in individual Microsoft Word documents. The participant responses were deidentified to maintain patient confidentiality.

### Data Analysis

The qualitative data from the semistructured interviews were analyzed using thematic analysis, a 6-phase method for organizing, identifying, and summarizing patterns and themes [24]. In the first phase, known as data familiarization, researchers carefully reviewed, extracted, and organized the textual data in Microsoft Excel. The second phase involved generating initial codes for the data using a grounded theory approach, which explores participants’ attitudes, beliefs, norms, and processes to develop hypotheses from the data rather than testing preexisting hypotheses [25]. In the third phase, the assigned codes were aggregated to uncover underlying patterns, themes, and subthemes. Phases 4 and 5 focused on reviewing, refining, and defining these themes, subthemes, and codes. Finally, in phase 6, the results of the thematic analysis were summarized and reported.

A sample size of 20 participating patients was targeted in accordance with rule-of-thumb guidance for a pilot study emphasizing qualitative assessment and protocol feasibility [26]. Descriptive statistics of participants and nonparticipants in VR were used to describe the population of interest. For physiologic signals, the first 2 minutes of pre-VR recording were compared to the first 2 minutes after the VR experience. Kubios automatic beat detection software was used to preprocess the heart rate data [27]. Heart rate variability was characterized by the square root of the Baevsky stress index (SI) [28]. Pre-post comparisons of vital sign data and mood assessments were performed using paired 2-sided *t* tests. Statistical analyses were performed using Stata version 18 (StataCorp) and the code is openly available on GitHub [29].

## Results

**Figure 3:**
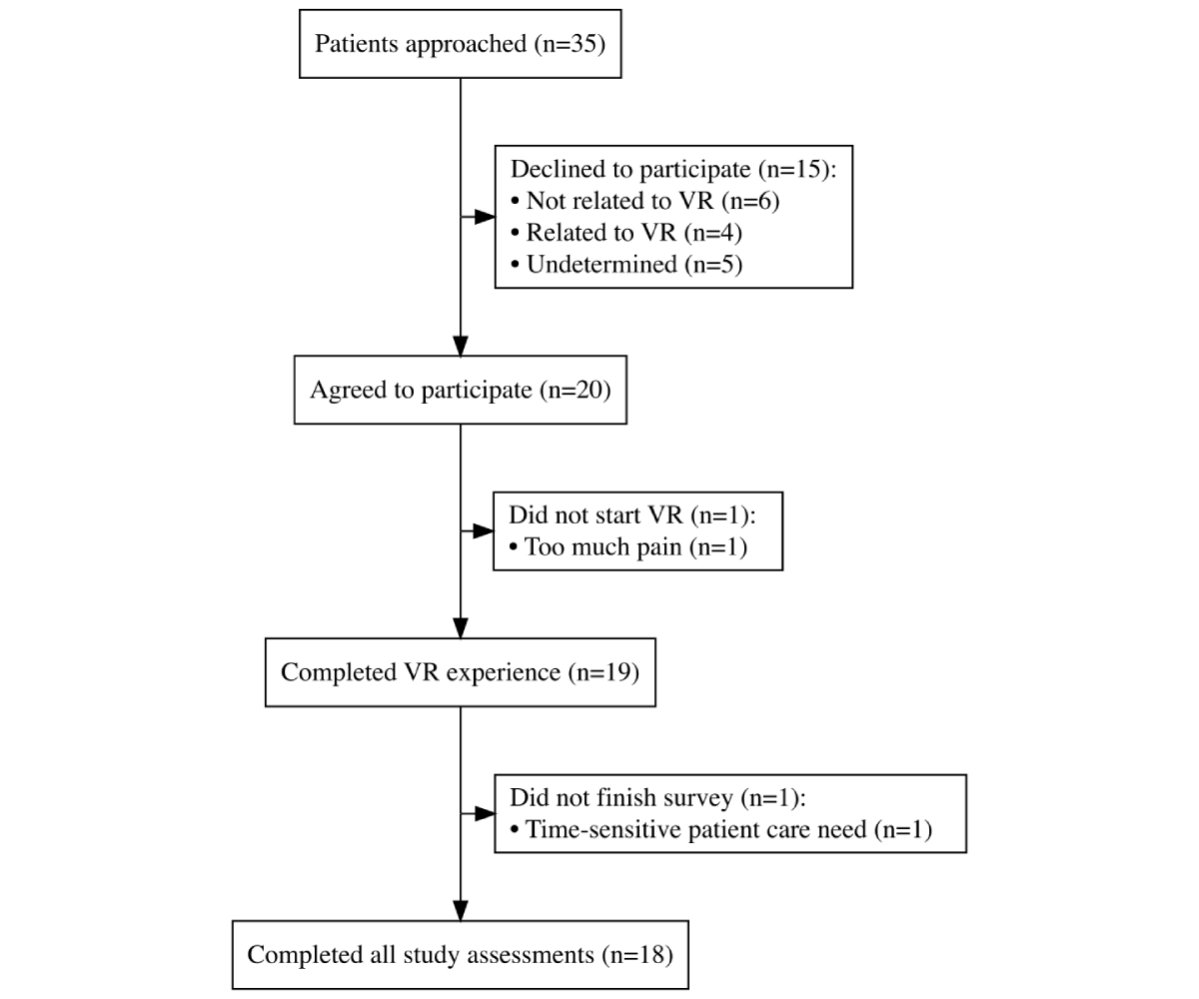
Enrollment flow diagram. VR: virtual reality.

Patients were enrolled between November 8, 2023, and February 6, 2024. A total of 35 patients were identified as potential candidates and approached, 20 (57%) of whom agreed to participate in the VR experience (Figure 3). Comorbidities and reasons for ICU admission mirrored those of the general ICU population (Table 1).

Characteristics of participants and nonparticipants are listed in Table 2. The age of participants (mean 61, SD 17 years) did not differ significantly (*P*=.33) from that of nonparticipants (mean 54, SD 22 years); however, nonparticipants were significantly more likely to have previously used VR (4/15 vs 1/20; *P*=.005).

Among the 20 patients consenting to participate, 19 completed at least 5 minutes of the VR experience (1 did not begin VR due to uncontrolled pain, but completed the pre-VR baseline data collection) and 18 completed all study assessments (1 had competing care needs prior to the interview). Participants used VR for a mean of 10 (SD 3) minutes. Among the 19 participants who completed the experience, 10 (53%) chose the travel experience, 5 (26%) chose the nature experience, and 4 (21%) chose the synthetic experience. No cybersickness or other adverse events occurred.

Among the 17 patients with valid heart rate data (1 patient had an excess artifact), the mean heart rate prior to initiation of the VR experience was 86.1 (SD 11.8) beats per minute (bpm), which decreased by 1.1 (95% CI 0.3-1.9; *P*=.008) bpm by the end of the experience. Mean heart rate variability, based on the SI, was 40 (SD 23) seconds^-2^ at baseline and decreased by 5.0 (95% CI 1.5 to 8.5; *P*=.008) seconds^-2^.

**Figure 4:**
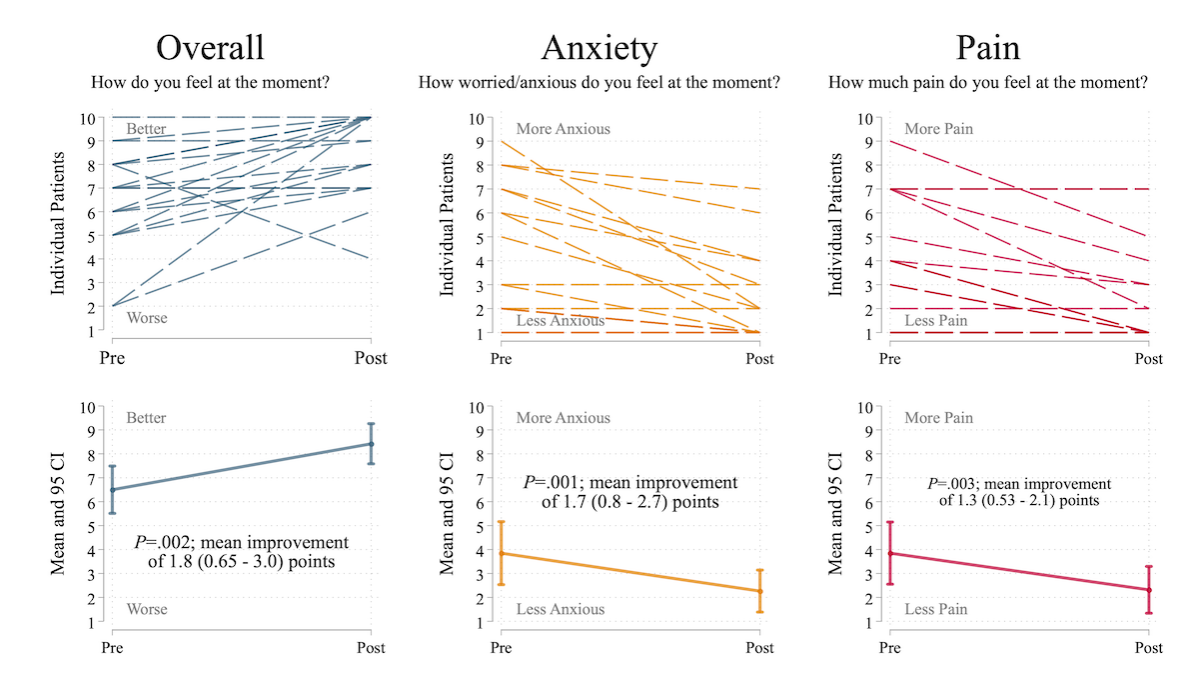
Overall mood, anxiety, and pain scores before and after use of immersive virtual reality. All scores were assessed using visual analog scales (see Multimedia Appendix 2) on a 1-10 scale, with 10 being best for overall mood and 1 being best for anxiety and pain. The paired t test was used for comparisons and 95% CIs of the mean change are presented in parentheses.

At baseline, participating patients reported moderate overall well-being (mean 6.5, SD 2.1 on a 1-10 visual analog scale with 10 being the best; n=20), anxiety (mean 4.0, SD 2.8 with 1 indicating no anxiety; n=20), and pain (mean 3.9, SD 2.8 with 1 indicating no pain; n=20). Overall mood improved by a mean of 1.8 points (95% CI 0.65-3.0; *P*=.002; n=19) from baseline, anxiety decreased by 1.7 points (95% CI 0.8-2.7; *P*=.001; n=19), and pain decreased by 1.3 points (95% CI 0.53-2.1; *P*=.003; n=19) after use of immersive VR (Figure 4).

**Table 1:** Characteristics of patients approached (N=35).

Characteristics		Value	
Age (years), mean (SD)		58 (19)	
Female, n (%)		11 (31)	
**Comorbidities, n (%)**			
	Type 2 diabetes	12 (34)	
	Atrial fibrillation	6 (17)	
	Chronic obstructive pulmonary disease	3 (9)	
	Congestive heart failure	7 (20)	
	Obstructive sleep apnea	2 (6)	
	Chronic kidney disease	3 (9)	
	Deep vein thrombosis	2 (6)	
	Cirrhosis	5 (14)	
	Cancer (any)	5 (14)	
**Common reasons for ICU** ^a^ **admission, n (%)**			
	Respiratory failure		10 (29)
	Glucose/electrolytes		4 (11)
	Thromboembolism		3 (9)
	Gastrointestinal bleed		3 (9)
	Heart failure		2 (6)
	Sepsis		2 (6)

^a^ICU: intensive care unit.

**Table 2:** Characteristics of patients who declined or agreed to participate in the virtual reality (VR) experience.

Characteristics			Declined (n=15)		Agreed (n=20)		*P* value
Age (years), mean (SD)			54 (22)		61 (17)		.33
Female, n (%)			6 (40)		5 (25)		.34
**Race/ethnicity, n (%)**							.40
	Asian	0 (0)		1 (5)			
	Black	0 (0)		1 (5)			
	Native Hawaiian/Pacific Islander	1 (9)		0 (0)			
	White	10 (91)		18 (90)			
	Hispanic	3 (27)		3 (15)		.41	
Hearing impairment, n (%)			1 (11)		0 (0)		.13
Wears eyeglasses, n (%)			4 (44)		9 (45)		.98
**Respiratory support, n (%)**							.32
	Face mask or variant	1 (9)		0 (0)			
	High-flow nasal cannula	1 (9)		6 (30)			
	Nasal cannula	5 (45)		9 (45)			
	None	4 (36)		5 (25)			
Prior VR use, n (%)			4 (57)		1 (6)		.005

Five themes were commonly mentioned in the post-VR interviews (Table 3). Use of the VR headset to view relaxing scenery content was met with approval by all patients. Patients had a variety of positive responses with use of the VR headset, which was described as “good,” “easy,” “enjoyable,” “comfortable,” and “pleasant.” These responses indicate overall acceptance and satisfaction.

Patients also identified potential benefits of VR in alleviating symptoms of anxiety and depression. Many reported a reduction in feelings of anxiety, nervousness, and isolation, suggesting a positive impact on mental well-being. Several patients highlighted that the VR intervention helped distract them from their current pain symptoms. The immersive nature and engaging VR content enabled patients to briefly “forget” about their pain and discomfort. The VR environment was described by patients as “calming,” “relaxing,” and “meditative.” The opportunity to escape the ICU environment and immerse themselves in nature or travel scenery was highly valued, contributing to a sense of relaxation and calm.

Lastly, most patients reported having no problems using the VR headset. A few patients reported experiencing slight discomfort with the headset weight and difficulty seeing the side visuals. No patients reported motion sickness or claustrophobia.

**Table 3:** Themes and representative quotes from interviews after immersive virtual reality (VR) use among patients in the intensive care unit.

Theme	Representative quote
Acceptance of the VR system	“It was comforting, it was easy, from start to finish it’s so calm, it felt so fast. I don’t mind wearing it longer. it was my first time using it, I was amazed.” [P18]
Improvement in mental health symptoms	“I think will help someone like me, facing what I face, sitting here for all days, waiting for my surgery, being anxious, not having anything to do, and not being allowed to eat or drink. I really appreciate you guys showing up. It really helps me.” [P20]
Distraction from pain symptoms	“I forgot about the pain, some of it is there. I feel relaxed and comfortable, it took me out of my mind and I’m able to focus on the virtual world, I like what I see, it’s so beautiful.” [P09]
Feelings of relaxation, calmness	“Mostly the calmness, being calm helps you deal with your physical issues better, and the visual experience has a lot of benefits, it helped me forget that I have clog in my lung.” [P16]
Problems using the VR headset	“No, don’t have any problem with it, it could be lighter with the headset. The side part it’s a bit blurry, but the other it’s good. It will be good to wear my glasses.” [P12]

## Discussion

### Principal Findings

In this feasibility study of commercially available immersive VR use among critically ill patients, we found that most patients did not have prior experience with VR technology but had high levels of interest and acceptance of the technology. Participants commented on VR’s potential to alleviate cognitive and emotional symptoms. Changes in heart rate variability were consistent with increased relaxation. Only minor technical challenges and no severe adverse effects were noted.

### Comparison to Prior Work

Prior work has explored the potential for immersive VR for several ICU use cases [1]. However, this previous work reported widely variable rates of uptake and hinted at a “digital divide,” where older patients may be less interested in new technology [30]. In contrast, we found high levels of participation among patients of all ages. Thus, VR might be considered for study even in settings that frequently care for older adults. We found that patients who had previously used VR were less likely to participate in our study. However, this might indicate that the novelty of the experience drove participation in this feasibility study. Interest may improve in prior users if VR were offered as a validated treatment. Only a minority of patients who did not participate gave reasons related to the VR itself.

After use, several participants commented on the potential of the therapy to address anxiety and foster relaxation or calmness. This was corroborated by the improvement in vital sign correlates of relaxation, such as improved heart rate variability. Some [6], but not all [18], prior studies of VR have shown consistent changes in vital signs.

Participants also highlighted the potential of VR to distract from pain, which is consistent with current guidance from the Society of Critical Care Medicine that recommends consideration of “cybertherapy [VR]” for this purpose [31]. The high usability scores and low rate of technical challenges with “off-the-shelf” commercially available options suggests that extensive customization is not a prerequisite for use. Furthermore, we did not observe claustrophobia, nausea, or “cybersickness” in any patients. Cybersickness may be less common with more modern VR headset technology that minimizes latency [32] and discordance between virtual and actual head positioning [33], which could explain why this was not encountered in our study. In contrast to prior work suggesting that nature scenes may maximize relaxation [15], travel was the most frequent VR experience choice among our participants. The potential for VR to enable “escape” from the ICU was also frequently mentioned in qualitative interviews.

### Strengths and Limitations

Strengths of this study include the assessment of both quantitative and qualitative dimensions of the VR experience, which both support the feasibility and potential utility of using VR in this group. In addition, there were few exclusion criteria and thus the sample of patients is expected to broadly represent characteristics of critically ill patients without delirium. Lastly, we demonstrated and reported an off-the-shelf and replicable experimental setup that investigators can use as a starting point for future studies.

However, several limitations also deserve mention. First, nonrepresentative sampling of research participants may have occurred due to only enrolling patients during select times when study staff were available. Despite exhaustive screening of patients who were identified as potential candidates during these times, we cannot fully describe what characteristics may have influenced which patients were judged to be potential candidates for the study by attending physicians. Second, there was no control group. Time trends and the influence of conversing with study staff may also contribute to changes in symptoms and vital signs, although we attempted to stabilize physiologic trends with a 5-minute run-in period and structured interview guides were used to focus the conversation. Third, prior work suggested that VR’s effectiveness correlates with the degree of immersion [34], which we did not directly assess. However, we chose not to attempt to eliminate distractions to better emulate the conditions and degree of immersion expected in actual use. Fourth, our quantitative outcome scales are slightly modified from previously validated work, and thus the reported effect sizes should not be compared to other interventions or established minimally important differences. Lastly, the uptake of VR may differ when it is offered as a treatment for specific conditions as opposed to offering the opportunity to help assess feasibility, particularly among patients who have previously used the technology.

### Future Directions

This work has several important implications for the study of VR in the ICU. We used commercially available technology and found that the sessions were acceptable to patients. This suggests that customization of either software or hardware is not necessarily required for some VR use cases. Furthermore, we found high interest among critically ill patients. Lastly, we encountered minimal disruptions to patient care or study protocols, with 95% of the patients completing the experience and 90% completing all study assessments. This suggests that protocols to study the impact of VR can be integrated into usual ICU care with little impingement on clinical workflows. Evaluation of the usability in patients excluded from the current work, such as those with mild delirium or more severe emotional symptoms, could help establish the potential of studying immersive VR in additional high-risk populations.

### Conclusions

We found that a relatively short session of off-the-shelf immersive VR is acceptable to critically ill patients; resulted in improved pain, anxiety, and overall mood scores; and did not result in side effects or present major technical challenges. This work suggests that studies on the effect of VR on patient-relevant outcomes in the context of critical illness are feasible. Investigators can consider VR study protocols that do not involve substantial technology customization or large changes to patient care workflows.

## Appendix

Multimedia Appendix 1 STROBE (Strengthening the Reporting of Observational Studies in Epidemiology) checklist for reporting of observational research.

Multimedia Appendix 2 Visual analog scales used for assessing mood, anxiety, and pain before and after the virtual reality experience.

Multimedia Appendix 3 Interview guide for the qualitative assessment after the virtual reality experience.

## Abbreviations

bpm: beats per minute
ICU: intensive care unit
SI: stress index
STROBE: Strengthening the Reporting of Observational Studies in Epidemiology
VR: virtual reality

## Data Availability

Data can be provided upon Institutional Review Board–approved request to Joseph.Finkelstein@utah.edu.
