# Supplementary material for: Immersive Virtual Reality Use in Medical Intensive Care: Mixed Methods Feasibility Study
Source: JMIR Serious Games. 2024 Aug 9;12:e62842. doi: 10.2196/62842 (PMC11344185; doi:10.2196/62842)

Study ID__________ **RA_____________________** **Date__________________**

Question 1

Please describe how you feel at the moment?

1 means very bad and 10 means fantastic.

1 2 3 4 5 6 7 8 9 10

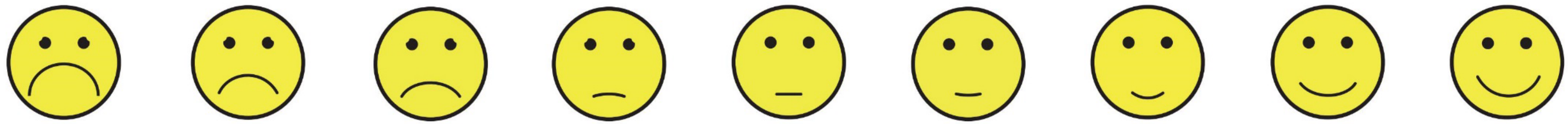

Study ID__________ RA____________________ Date__________________

Question 2

Please describe how worried/anxious you feel at the moment?

1 means not worried/anxious and 10 means extremely worried/anxious.

1 2 3 4 5 6 7 8 9 10

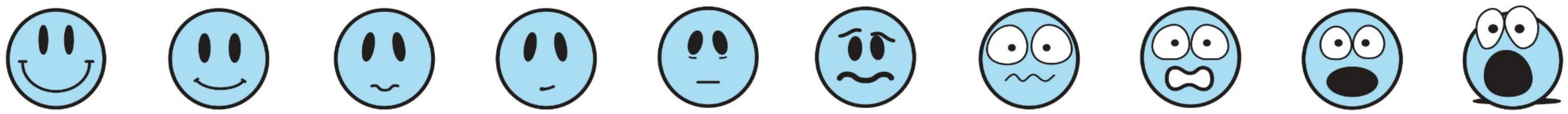

Study ID__________ RA____________________ Date__________________

Question 3

Please describe how much pain you have at the moment?

1 means you have no pain and 10 means you have the worse pain ever.

1 2 3 4 5 6 7 8 9 10

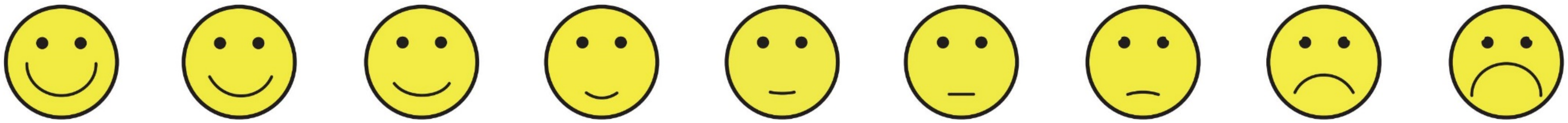

Supplement: Multimedia Appendix 2 [file games_v12i1e62842_app2.pdf]
