# Supplementary material for: Immersive Virtual Reality Use in Medical Intensive Care: Mixed Methods Feasibility Study
Source: JMIR Serious Games. 2024 Aug 9;12:e62842. doi: 10.2196/62842 (PMC11344185; doi:10.2196/62842)
Supplement: Multimedia Appendix 3 [file games_v12i1e62842_app3.pdf]

Before setting up the virtual reality headset, the patient will be presented with a visual-analog scale that asks them to point to the face (frowny to happy) indicating their current overall mood, anxiety, and pain levels. [see attached document]

They will experience the VR headset for 5-15 minutes; with vital sign recording.

We will again have them indicate their mood, anxiety, and pain levels on the same scale.

Then, we will perform a qualitative interview based on the following thematic questions:

1. Please describe your experience using the VR headset
2. In what ways did this help you feel better?
3. What problems did you have using this headset?
4. How did this compare to what your initial expectations?
5. How do you think we could use this in the future with other patients?

We will refine each interview question based on the feedback from each interview, including further assessment of any themes that participants bring up.

«Image:Stamp» «Institution»
«IRB»
«Approved» «ApprovedDate»
«Expiration» «ExpirationDate»
«Number»
